# PARG suppresses tumorigenesis and downregulates genes controlling angiogenesis, inflammatory response, and immune cell recruitment

**DOI:** 10.1186/s12885-022-09651-9

**Published:** 2022-05-18

**Authors:** Sarah Johnson, Yaroslava Karpova, Danping Guo, Atreyi Ghatak, Dmitriy A. Markov, Alexei V. Tulin

**Affiliations:** 1 Department of Biomedical Sciences, School of Medicine and Health Sciences, University of North Dakota, Grand Forks, ND 58202 USA; 2 Koltzov Institute of Developmental Biology of Russian Academy of Sciences, Moscow, 119334 Russia; 3 Department of Cell Biology and Neuroscience, School of Osteopathic Medicine, Rowan University, Stratford, NJ 08084 USA

**Keywords:** PARP, PARG, Poly(ADP-ribose) glycohydrolase, Poly(ADP-ribose) polymerase, Poly(ADP-ribose), chemokines, Tumorigenesis, 3 T3 cells, Doxycycline

## Abstract

Chemokines are highly expressed in tumor microenvironment and play a critical role in all aspects of tumorigenesis, including the recruitment of tumor-promoting immune cells, activation of cancer-associated fibroblasts, angiogenesis, metastasis, and growth. Poly (ADP-ribose) polymerase (PARP) is a multi-target transcription regulator with high levels of poly(ADP-ribose) (pADPr) being reported in a variety of cancers. Furthermore, poly (ADP-ribose) glycohydrolase (PARG), an enzyme that degrades pADPr, has been reported to be downregulated in tumor tissues with abnormally high levels of pADPr. In conjunction to this, we have recently reported that the reduction of pADPr, by either pharmacological inhibition of PARP or PARG’s overexpression, disrupts renal carcinoma cell malignancy in vitro. Here, we use 3 T3 mouse embryonic fibroblasts, a universal model for malignant transformation, to follow the effect of PARG upregulation on cells’ tumorigenicity in vivo. We found that the overexpression of PARG in mouse allografts produces significantly smaller tumors with a delay in tumor onset. As downregulation of PARG has also been implicated in promoting the activation of pro-inflammatory genes, we also followed the gene expression profile of PARG-overexpressing 3 T3 cells using RNA-seq approach and observed that chemokine transcripts are significantly reduced in those cells. Our data suggest that the upregulation of PARG may be potentially useful for the tumor growth inhibition in cancer treatment and as anti-inflammatory intervention.

**Supplementary Information:**

The online version contains supplementary material available at 10.1186/s12885-022-09651-9.

## Introduction

The poly(ADP-ribosyl) ation pathway (also known as parylation) regulates many cellular processes by re-modeling chromatin, including transcription, stress response, DNA repair among others [1–3]. The cellular level of poly(ADP-ribose) (pADPr) depends on the relative activity of two counteracting enzymes: poly(ADP-ribose) polymerase (PARP), which modifies proteins by adding poly(ADP-ribose) moieties to their surface, and poly(ADP-ribose) glycohydrolase (PARG), which degrades pADPr polymers [4]. The main PARP in mammal cells is PARP1 – nuclear enzyme that is highly enriched in chromatin and was shown to be associated with promoter of genes [5–7]. The synthesis of pADPr, the long branched nucleic acid, twice more negatively charged than DNA, repulse other chromatin proteins from DNA in dependent genes areas and make it accessible to other binding factors that regulates transcription [5]. The intensive studies on revealing PARP1 dependent genes are ongoing and while some of them, such as heat shock and NF-kB target genes, are already identified, many others are still to be determine [5, 8, 9]. Moreover, it is known that these targets sometimes demonstrate particular cell state and type variabilities [10].

The mode of action for pADPr on cellular processes is quite complex and depends on the rate of poly(ADP-ribosyl) ation, which protein factors and chromatin loci are involved, as well as the level of self-activation of PARP through the attachment of poly(ADP-ribosyl) residues and other regulatory modifications the two enzymes may undergo [11]. Since many cancer cells have been found to accumulate abnormally high levels of pADPr [12], the dysregulation of poly(ADP-ribosyl) ation pathway has been implicated in tumorigenesis, and thus has been a point of interest for the development of new cancer treatments [13, 14]. While molecular mechanisms regulating the activity of PARP proteins have been exhaustively scrutinized [4, 15], PARG has not yet been intensively studied due to its low cellular content, the lack of cell-applicable, PARG-specific chemical inhibitors, as well as reliable PARG genetic models [16]. Both PARP and PARG have been shown to localize to the same loci with elevated levels of pADPr [17].

Consequently, despite the fact that PARG has an opposite to PARP activity and may be approached as a means to downregulate pADPr, the majority of published data on PARG focuses on its inactivation **[**18**–**21**]**, and, in addition to PARP inhibitors, a number of PARG inhibitors have recently emerged as potential anti-cancer interventions **[**13**,** 15**,** 22**,** 23**]**, linking the therapeutic effect of PARG inhibitors with the deficiencies in DNA replication **[**24**–**26**]**. Since PARP is known to poly(ADP-ribosyl) ate itself as a mean of self-inactivation **[**27**]**, in those cancers where PARG’s upregulation correlates with poor survival rate, an elevated level of PARG may be sufficient to keep PARP re-activated but, at the same time, may not be sufficient to reduce the overall level of pADPr and thus to suppress tumorigenicity. While the physiological effect of inhibitors may, in fact, depend on the ratio between these two counteracting enzymes in a particular cancer type, the most recent statistical analysis of long-term renal cancer patients’ survival indicates that PARP1 remains “prognostic, high expression is unfavorable in renal cancer”, but, at the same time, PARG appears to be not prognostic in renal cancer, and its increased density correlates with patients’ survival (Supplemental Fig. S1). Following these studies, our recently published data on clear cell renal cell carcinoma (ccRCC) demonstrated that upregulation of PARG reduces the clonogenic activity ccRCC cells in vitro, which is also associated with downregulation of genes associated with a number of tumorigenic pathways **[**28**]**. Thus, although PARG inhibition may be useful for treatment of certain cancers due to their complex etiology, the upregulation of PARG, which may be more straightforward and effective in other cancers, have not been thoroughly studied in vitro and in vivo. And yet, very little is known about the mechanisms of PARG regulation, its role in tumorigenesis, and molecular pathways that might be affected by PARG’s inhibition or activation in metastatic tumors.

Addressing the problem of mapping pADPr-associated proteome and PARG targets is complicated by the transient nature of pADPr moieties, the complex biochemical behavior of pADPr-modified proteins in vitro, and the presence of seventeen PARP-related genes in mammalian genomes [29]. To date, multiple papers have identified a set of pADPr-responsive factors that regulate global gene expression. PARP-1, the major isoform of PARP in mammals, has been demonstrated to be a cofactor of various transcription factors and chromatin-associated proteins, including NFkappaB, Activator Protein 1 (AP-1), histones, and an activator of different regulatory pathways (Wnt, SIRT-1) [20, 30–33]. In addition, poly(ADP-ribosyl) ation pathway has been implicated in promoting inflammatory responses by inducing cytokines such as TNFα and cell adhesion molecules [34–38]. Elevated levels of pADPr have also been associated with the release of various chemokines, including monocyte chemoattractant protein-1 (MCP-1 or CCL2), eotaxin (CCL11), macrophage inflammatory proteins MIP-1α (CCL3) and MIP-1β (CCL4), all of which are known to promote cancer invasion [39]. Chemokine expression triggers the degradation of extracellular matrix by matrix metalloproteinases (MMPs), the endothelial cell migration, and the induction of angiogenic pathways such as the vascular endothelial growth factor (VEGF) pathway. And yet, while the expression of chemokines has been directly implicated in every stage of cancer development [40, 41], the role of PARG in controlling cellular chemokines remains elusive and controversial. The inhibition of PARG has been shown to mediate post-traumatic inflammatory reaction [42] and to induce the expression of proinflammatory genes by macrophages [43]; on the other hand, the decrease of PARG’s activity has been demonstrated to reduce septic shock in mice [44] and to protect animals against renal ischemia/perfusion injury [45]. To date, despite the fact that PARG was discovered during early studies on DNA damage, inflammation and tumorigenesis, the role of PARG in controlling gene expression in tumors, especially in respect to its pADPr-degrading activity, has not been sufficiently explored.

**Fig. 1:**
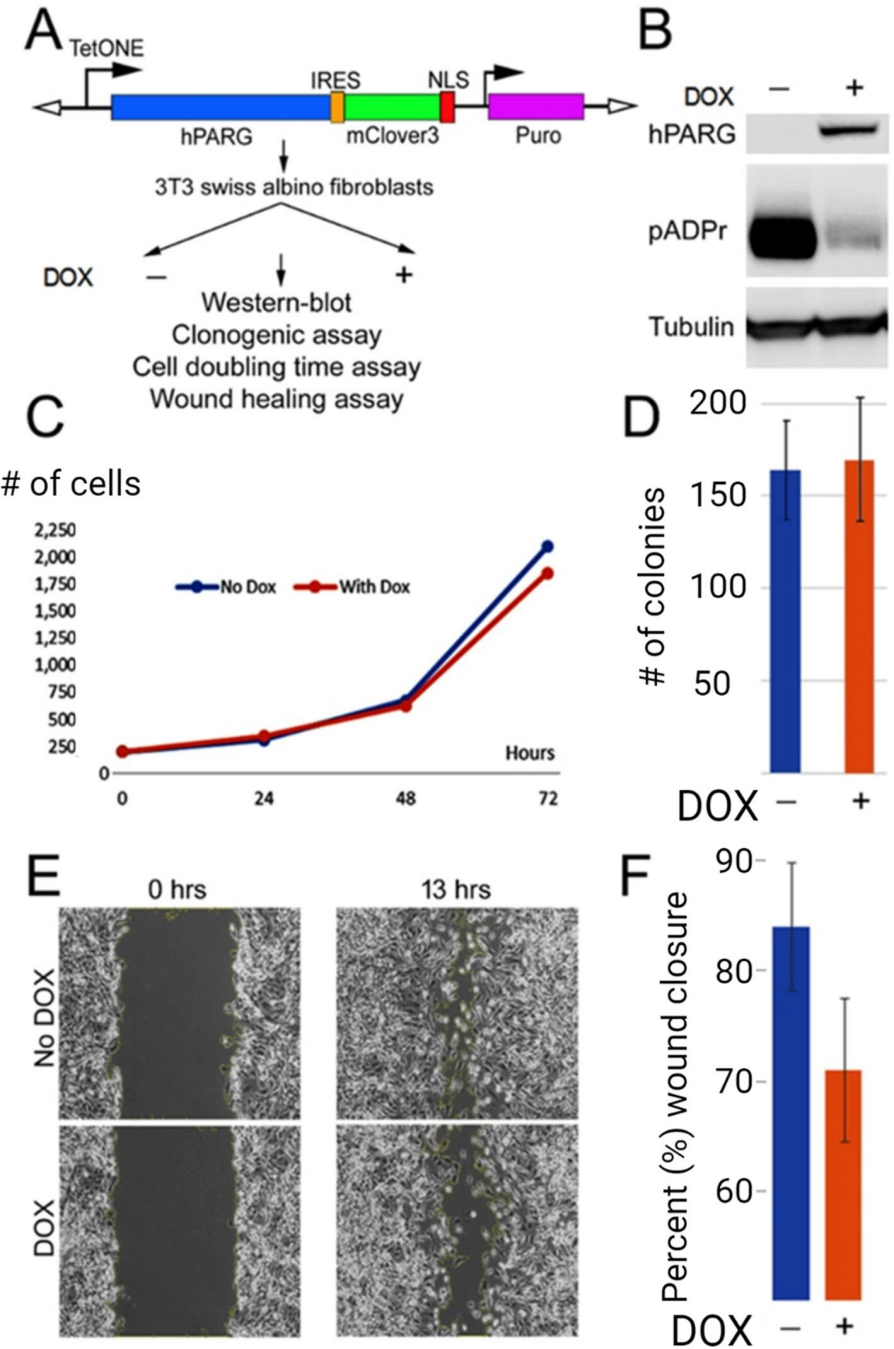
The ectopic expression of hPARG in 3 T3 cells does not affect their viability in vitro. **A** Schematic of the 13.5 kb lentiviral cassette pLVX-TetONE-hPARG-IRES-mClover3-NLS-Puro co-expressing hPARG and nucleus-targeted fluorescent protein mClover3 under control of the TetON promoter. The cassette was used to generate a 3 T3 cell line which stably expresses hPARG in the presence of doxycycline (3 T3-hPARG). The Puromycin-resistant gene (Puro) was used as a selection marker. The cells were split into two test groups, one of which received 500 ng/ml doxycycline (+) and the other receiving no doxycycline (−). These cells were then subjected to the assays indicated. **B** Rabbit monoclonal anti-hPARG and mouse anti-pADPr antibodies were used to detect the hPARG polypeptide and pADPr moieties, respectively, in 3 T3-hPARG cells that were cultured either in the absence (−) or in the presence (+) of doxycycline. Staining with anti-tubulin antibodies was used as a loading control (Tubulin). The uniform expression of hPARG in cultured cells and the corresponding reduction in pADPr was confirmed by immunofluorescence (Supplemental Fig. S4). Uncropped version of Western Blot is presented at Supplemental Fig. S5. **C** Doubling time assay comparing 3 T3-hPARG cell cultures grown either in the presence (‘With Dox’; red line) or in the absence (‘No Dox’; blue line) of doxycycline with regard to the number of cells produced over the indicated time periods. The two treatment conditions showed a similar doubling time (26 ± 7 hours). **D** Clonogenic assay comparing # of clones (≥50 cells/each) formed by single 3 T3-hPARG cells over 14 days either after doxycycline induction (+DOX) or in the absence of doxycycline (−DOX) (*n* = 3 for each group; error bars represent SEM). **E** Wound healing assay shows the ability of cultured 3 T3-hPARG cells to restore the damaged monolayer in the presence (DOX) and in the absence of doxycycline (No DOX). **F** 3 T3-hPARG wound healing assay quantified as % of wound closure (*n* = 5; the difference is not statistically significant (t-test *p* value = 0.2076). Error bars represent SEM

Human PARG (hPARG) is encoded by a single gene as its mammalian paralogs. Previously, we demonstrated that the controlled overproduction of hPARG in cultured clear cell renal cell carcinoma (ccRCC) cells reduces their clonogenic activity in vitro, which was associated with the downregulation of key cancer-related genes [28]. Similarly, we used PARG-overexpressing cells to model the activation of PARG by increasing the cellular pool of active PARG polypeptide. The isolation of stably expressing cell lines from patient-derived tumors is often hindered by significant genetic variations among isolates and overall genetic instability, including chromosomal aberrations. To circumvent this, here we utilize allogenic NIH/3 T3 Swiss albino embryonic fibroblasts (3 T3 cells), a universal system for studying malignant transformation of highly proliferative cells [46–48], to stably express hPARG in grafted cells using previously published lentiviral hPARG-TetON system [28] (Fig. 1, panel A). hPARG coding sequence is introduced under TetON promoter that starts to be expressed when permanently lentivirus-transduced 3 T3 cells are treated with doxycycline. Therefore, doxycycline treatment will induce hPARG overexpression in transduced 3 T3 cells, while will not affect the PARG level in untransduced cells [49, 50]. To follow alterations in the expression pattern of cancer-associated genes induced by the change in PARG level, we use the utility of 3 T3 cells and RNA-seq approach to compare transcription profiles of transfected tumor cells and their immediate precursors. The analysis of our RNA-seq data reveals that the expression of pro-tumor cytokines and chemokines involved in the promotion of angiogenesis, tumor growth, and inflammatory response is significantly downregulated in the cells expressing hPARG relative to the control group. The detail data presented below may pave a new path for utilizing the poly(ADP-ribosyl) ation pathway components for the treatment of cancers and associated inflammatory responses, as well as for therapeutic prevention of oncogenesis.

## Materials and methods

### Cell culture

Albino Swiss Mouse Embryonic Fibroblasts (3 T3 line or 3 T3 cells) were acquired from Nexus Pharmaceuticals, Inc. Cells were grown in RPMI1640 with medium supplemented with 10% (v/v) inactivated fetal bovine serum (FBS) (Atlanta Biological, R&D Systems, CAT# S11150H) and 100 units/ml penicillin G sodium, 100 μg/ml streptomycin sulfate (Gibco, CAT#15240062) and 1X MEM non-essential amino acids (Gibco, CAT# 11140050). Human lenti-X 293 T cells (Clontech/Takara, CAT# 632180) were maintained in 90% DMEM (Sigma, CAT# D5796) with 10% (v/v) inactivated fetal bovine serum (FBS), 100 units/ml penicillin G sodium, and 100μg/ml streptomycin sulfate, and used as the host cell line for lentiviral packaging. Human HT1080 (ATCC, CAT#CCL-121) cells for lentiviral titration were grown in EMEM (ATCC, CAT# 30-2003).

### Plasmids and vectors

The lentiviral construct, pLVX-TetOne-hPARG-IRES-mClover3-NLS-Puro is based on pLVX-TetOne-Puro vector system (Clontech/Takara, CAT# 631847) and allows the expression of human PARG gene under control of Tet-inducible promoter (TetONE) and nucleus-targeted fluorescent protein mClover3 ORF for the visual control of promoter activity. The detail information on this construct and its complete sequence has been published previously [28].

### Packaging Lenti-X vectors into lentiviral particles and virus tittering

The lentiviral construct, pLVX-TetOne-hPARG-IRES-mClover3-NLS-Puro was packaged into lentiviral particles using Lenti-X Packaging Single Shots Protocol from Clontech/Takara. Lentiviral vector stocks were collected and concentrated using the Lenti-X Concentrator Protocol (Clontech/Takara). Lenti-X virus stocks were tittered using puromycin selection with HT1080 cells; the titer of virus corresponded to the number of colonies generated by the highest dilution multiplied by the dilution factor.

### Single clones screening and transduction of selected clones for hPARG expression

2 × 10^5^ 3 T3 cells were cultured in 6-well plates in complete growth medium for 12-18 hr. before transduction. Polybrene was added to the cell culture to obtain a final concentration of 4 μg/ml to increase the efficiency of lentivurus transduction according manufacturer recommendation (Clontech/Takara). 3 T3 cells were transduced with a multiplicity of infection (MOI) of 30. The cells were transduced, and, after 24 hr. post-transduction, the medium was removed, and the cells were trypsinized and split. Cells from a single well of a 6-well plate were split into 4 10-cm dishes containing complete growth medium supplemented with 2.25 μg/ml of Puromycin. Puromycin-resistant colonies were transferred into separate wells of a 24-well plate and cultured in the presence of puromycin. To select colonies for hPARG expression, isolated colonies were split and further cultured either in the presence or in the absence of 500 ng/ml doxycycline for 72 hr. hPARG expression was determined by Western Blotting analysis and the fluorescent signal generated by mClover3 fluorescent protein (captured by Biotek Cytation 3 imaging reader). Clones with the highest fold change of hPARG induction vs control were selected for propagation and further testing.

### Cell culture experiments

3 T3 cells with confirmed expression of hPARG and their lentivirus-free controls were grown in complete RPMI-1640 media (Invitrogen) supplemented with 10% Fetal Bovine Serum (Atlanta Biologics), 1% of 100X Penicillin-Streptomycin (Gibco), non-essential amino acids (NEAA) (Gibco) and 2.5 μg/ml puromycin (Takara) to ensure the integrity of lentiviral construct. All cells were maintained in CO_2_ incubator at 37 °C with 5% CO_2_. To overexpress the gene of interest (hPARG), 500 ng/ml doxycycline (Takara) was added to the cells and maintained for 72 hrs. Control wells were maintained without doxycycline stimulation in the same medium for the same time period.

### Western blotting and immunohistochemistry

Polypeptides from total cell lysates were separated by SDS-PAGE and transferred to a PVDF membrane for analysis. All blots were blocked in 5% dry milk resuspended in 1X PBS (PBS Santa Cruz, CAT# sc-24,947), 0.1% Tween 20 (Sigma, CAT# P2287), then were probed by incubation with the following primary antibodies at 4 °C: rabbit monoclonal anti-hPARG (1:2000) (Cell Signaling Technology, CAT# D4E6X), mouse monoclonal anti-pADPr (1:150) (Santa Cruz Biotechnology, CAT# 10H), mouse monoclonal anti-Tubulin (1:20000) (Sigma, CAT# B512). Membranes were then washed 3 times with 1X PBST and subsequently incubated with corresponding secondary antibodies: HRP-labeled, anti-Rabbit IgG (Goat) for 30 minutes or HRP-labeled, anti-Mouse IgG (Goat) (both from PerkinElmer, 1:5000) for 45 min at RT. Immunoblot exposure was done by adding HyGlo chemiluminescent HRP antibody detection reagent (Fisher Scientific, CAT# NC9515009), and the imaging and densitometric analysis were conducted using a LI-COR Odyssey Fc imaging system (model 2800) and LI-COR Image Studio software v5.2.

### Cell doubling time

To check cell viability, 3 T3 cells with the lentiviral construct were plated in 6-well culture dishes with 2 × 10^5^ cells per well. An equal number of wells were assigned for the cells with doxycycline induction and the control group with no doxycycline added (no induction). Doxycycline induction (500 ng/ml) was maintained for 24, 48, and 72 hrs, at which time points induced and non-induced cells were counted to estimate the growth rate and the doubling time. At each time point, the cell number from each group was averaged, plotted on the graph, and the doubling time was calculated using the following formula:

$$\mathrm{Doubling}\;\mathrm{time}\;=\;\mathrm{duration}\;\ast\;\log\;\left(2\right)/\log\;\left(\mathrm{Final}\;\mathrm{concentration}\right)\;-\;\log\;\left(\mathrm{Initial}\;\mathrm{concentration}\right).$$

### Clonogenic assay

3 T3 cells were seeded in 6-well plates (200 cells/well). After 24 hrs, the plates were split into two groups, each of which received either 500 ng/ml doxycycline or vehicle as described for the Cell Doubling Time assay. The cells were grown for an additional 14 days with doxycycline and media being exchanged every 2 days. After crystal violet (0.5% w/v) staining, colonies of more than 50 cells were counted by the FIJI particle analyzer tool.

### Wound healing assay

For this assay, 2 × 10^5^ cells per well were seeded into 12-well plates and cultured in complete RPMI media as described earlier. An equal number of wells were kept as control and for the treatment group receiving 500 ng/ml doxycycline. Once cells became confluent in a monolayer, a wound was created by scratching the bottom of the well(s) with a 200 μl pipette tip in a gentle, steady, and straight motion. Pictures were taken immediately of the scratch area under a light microscope. This is considered the time point 0 (T0). Then the cell media were exchanged for reduced serum media (2% serum), and the cells were cultured with or without doxycycline, and the same exact wound areas were imaged after 12-18 hrs (Time ∆ h). Images were analyzed with Image J (imagej.nih.gov.ij/version 1.52a) wound healing tool (http://dev.mri.cnrs.fr/projects/imagej-macros/wiki/Wound_Healing_Tool). Averages of triplicates of control and treatment wells were taken for data analysis purpose. Percent (%) wound closure was measured by following formula [51]:

$$\left[\left( At=0\ h- At=\Delta h\right)/ At=0\ h\right]\times 100\%$$

### Animals for in vivo studies

Severely immunodeficient NSGTM mice (NOD.Cg-Prkdc<scid> Il2rg < tm1Wjl>/SzJ) were obtained from Jackson Lab, Bar Harbor, ME. All animals were maintained in standard housing conditions (12 hr. light/dark cycle, ad libitum food and water). All animal experiments were approved by the University of North Dakota Institutional Animal Care and Use Committee (IACUC). Animals with tumor allografts were euthanized according to IACUC-approved protocol (А1802-3) and before they reached the morbid state.

### Allografts

3 T3-hPARG cells were grown to 80% confluency, and 2 × 10^6^ cells were injected subcutaneously into the lower flank of 6-9 week-old female NSG mice. For the doxycycline experimental group, 3 T3-hPARG cells were stimulated with 500 ng/ml doxycycline for 72 hrs prior to the cell injection. All injected mice were observed closely for tumor growth. Once tumors started appearing, tumor volumes were measured by digital calipers every 2-3 days for up to day 36. Tumor volumes were calculated using the modified ellipsoid formula V = (length × Width^2)/2 [52]. The control groups of mice were maintained with regular diet while the experimental groups were supplemented with 200 mg/kg body weight doxycycline feed (Bioserve, NJ). Diet and water were refreshed every 5 days.

A parallel, control study was also performed using allografts with non-transformed 3 T3 cells. Tumor growth and development were followed in a similar way. These mice also received either control diet or a diet containing doxycycline as indicated.

### 3 T3 originated allograft

Allografts were dissected from mice and fixed in 4% paraformaldehyde (PFA) for 4 hour at 4C, submerged into 20% sucrose until sink and frozen in O.C.T. TissueTek (Fisher Scientific, Cat# 23-730-571). 12 μm sections were permeabilized with 0.3% TritonX-100 for 30 min RT and stained with anti-CD31 (1:500) (Abcam, ab28364) primary antibodies and goat anti-rabbit Alexa488 secondary antibodies (1:800) (Invitrogen, A-11008). Confocal images were taken on Leica DMI8 microscope.

### RNA-seq sample preparation

A 3 T3/pLVX-TetOne-hPARG-IRES-Puro hPARG-positive clone was grown in RPMI1640 (10% FBS, 100 units/ml penicillin G sodium, and 100 μg/ml streptomycin sulfate) with the addition of 2.5 μg/ml puromycin. The cells were split into six 10 cm dishes with 8 × 10^5^ cells per dish and incubated at 37 °C with 5% CO_2_ overnight. The following day the medium was exchanged to fresh complete medium with 2.5 μg/ml puromycin, with 3 dishes also receiving 500 ng/ml doxycycline. After 48 hrs, the medium was changed to fresh medium with 2.5 μg/ml puromycin, with 500 ng/ml doxycycline added to the same dishes. After 72 hrs, the cells were collected, and RNA was extracted according to the RNeasy protocol (Qiagen). The quality of RNA was determined by Bioanalyzer, the RNA integrity number for all RNA samples ranged 9.9-10. RNA samples were sent to Novogene for library preparation and RNA sequencing.

### Sequencing

RNA library preparation and 150 bp paired-end sequencing for mouse 3 T3 cells expressing human PARG via doxycycline induction was performed at Novogene Sequencing laboratory. mRNA was enriched via Poly(A) selection. Novogene libraries were prepared using NEB’s Ultra II RNA library kit and sequencing was performed on the NovaSeq 6000 system (Illumina). Illumina FQ sequencing files were imported into CLC Genomics Workbench version 12.0. The reads were trimmed to remove adapters then the trimmed reads were mapped to the GRCm38.96 *Mus musculus* genome using CLC’s default parameters (mismatch cost = 2, insertion cost = 3, deletion cost = 3, length fraction = 0.8, similarity fraction = 0.8, auto-detect paired distances, maximum number of hits per read = 10). All samples have a minimum of 50 million reads with 92% or greater mapping in pairs to the genome. At least 97% of the mapped reads mapped to genes. *N* = 3 for both treatment groups.

### Differential expression analysis and databases search

After mapping, differential expression for RNA-seq analyses was performed in CLC Genomics Workbench 12.0 on the gene-level expression tracks using whole transcriptome RNA-seq with TMM normalization. Differential expression due to treatment (hPARG+ or control) was tested with the comparison against the control group. Human Protein Atlas Database https://www.proteinatlas.org/ was accessed on 03/20/22. Survival plots were built at https://kmplot.com/ on 03/20/22.

### Quantitative PCR (qPCR)

Total RNA was extracted from cells using QIAshredder column and RNeasy kit (Qiagen), contaminating genomic DNA was removed by the g-column provided in the kit. cDNA was obtained by reverse transcription using M-NLV reverse transcriptase (Invitrogen). Relative quantitative PCR was performed using SYBR Green Master Mix (Bio-Rad) and ABI StepOne Plus real-time PCR system (Apply Biosystems, New York, NY, USA). qPCR was performed in 20 μl of master mix containing 10 μl SYBR, 100 ng cDNA and 250 nM of each forward and reverse primers. Primers used are listed in Table A2, Additional file 1 A**.** For all qPCR experiments, gene expression levels were normalized to the mouse housekeeping gene GAPDH and averaged from triplicates. The relative expression levels of the genes were measured using 2^-ΔΔCt method [53]. *N* = 3 biological replicates for each treatment group.

### Gene ontology analyses

Human orthologs for differentially expressed genes were determined by DIOPT (DRSC Integrative Ortholog Prediction Tool) score and enrichment tests were performed in IPA (Ingenuity Pathway Analysis) and Panther.

### Immunofluorescence staining

3 T3 cells were grown on 4 well Lab-Tek™ Chamber Slides (Thermo Fisher) with or without 500 ng/mL doxycycline for 72 h, washed with PBS, fixed with 4% PFA for 10 min, and permeabilized with 0.3% TritonX100 for 20 min at room temperature. Fixed cells were blocked in 5% normal goat serum (Thermo Fisher) on 0.1% TritonX100 and PBS for 1 h at room temperature and then stained with primary rabbit monoclonal anti-PARG antibodies (1:500, D4E6X, Cell Signaling) and mouse anti-pADPr antibodies (1:800, sc-56,198, Santa Cruz) on blocking solution at 4 °C overnight. Secondary anti-rabbit Alexa488 or anti-mouse Alexa568 antibodies (1:800, Invitrogen) diluted on blocking solution were used for final 1 h staining at room temperature. Slide wells were removed and cells were mounted into Vectashield mounting media (Vector Laboratories, San Francisco, CA, USA).

## Results

### The ectopic expression of hPARG in albino Swiss mouse embryo fibroblasts (3 T3 cells) significantly reduces the level of pADPr in these cells

To test whether PARG expression can influence tumorigenesis, we transfected mouse 3 T3 cells with a previously described lentiviral construct to express hPARG under doxycycline (DOX) control [28] (Fig. 1, panel A). The exposure of transfected cells (3 T3-hPARG) to doxycycline (+ DOX) resulted in a marked increase in hPARG polypeptide and a subsequent reduction of pADPr levels relative to the control (−DOX) (Fig. 1, panel B; a representative picture of treatment cell groups immunostained with anti-hPARG and anti-pADPr antibodies, and uncropped Western blots are shown on Supplementary Figs. S2 and S3, respectively) but did not affect the cells’ division rate and doubling time (Fig. 1, panel C). The lack of negative effect of pADPr reduction on cell viability was further confirmed by clonogenic assay (Fig. 1, panel D).

A standard in vitro wound healing or scratch assay was performed to study the coordinated movement of the cell population. Because cancer cells often show an aggressive migration pattern, we performed this assay to check whether doxycycline induced 3 T3-hPARG cells show a slower or altered rate of wound healing (Fig. 1, panel E). We observed an insignificant reduction in migratory activity among hPARG-expressing cells relative to the control (Fig. 1, panel F). Our experiments with ectopic expression of hPARG in cultured 3 T3 cells demonstrated that, although hPARG effectively degrades pADPr moieties, it does not affect the viability of the cells in vitro except for the slight reduction of migratory activity in the wound healing assay.

### The ectopic expression of hPARG in 3 T3 cells inhibits their tumorigenic potential in vivo

**Fig. 2:**
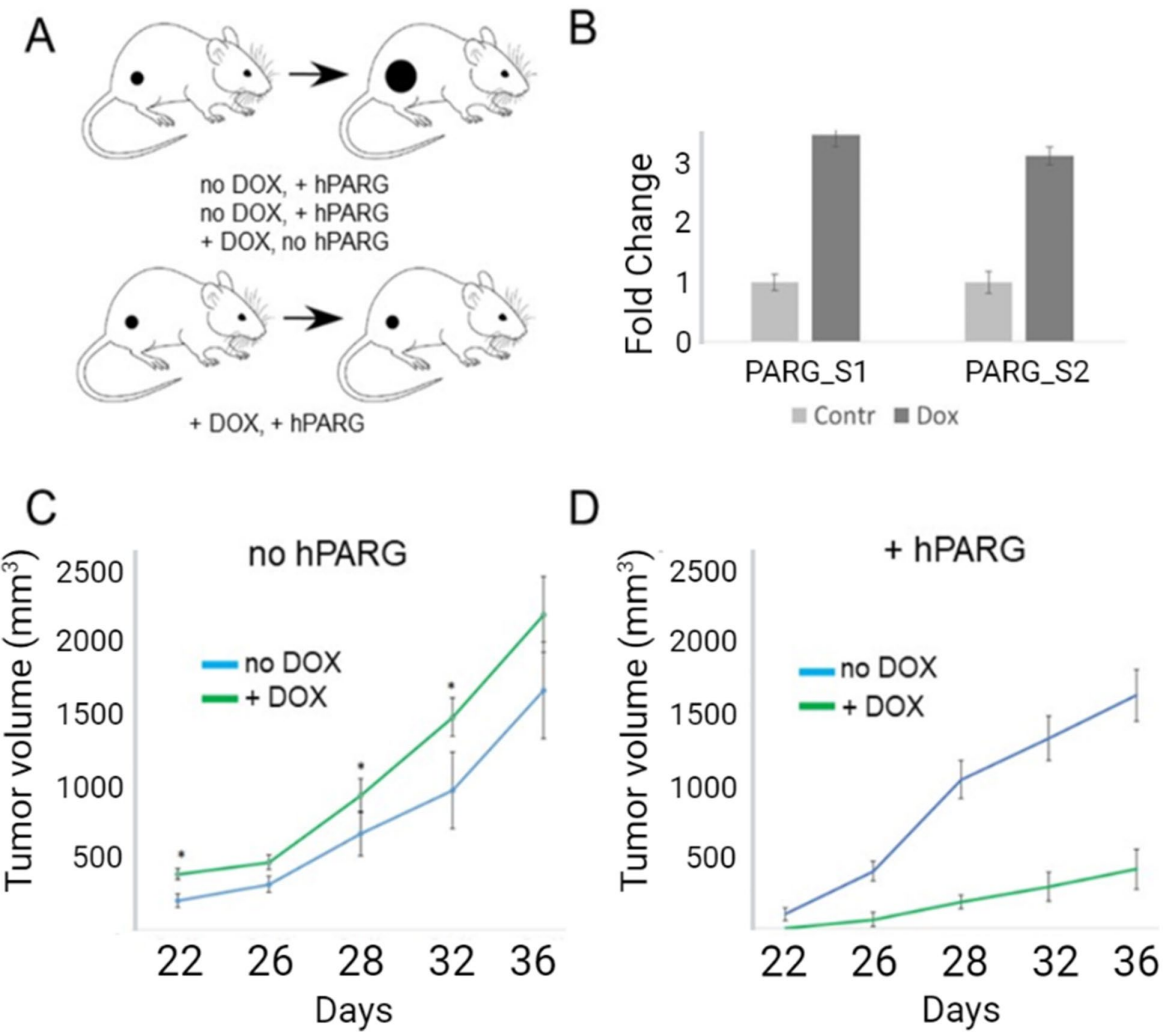
The ectopic expression of hPARG in 3 T3 cells inhibits their tumorigenic potential. **A** A 3 T3 cell-derived tumor allograft mouse model was used to test the effect of hPARG ectopic expression on tumorigenesis. Immunodeficient mice were split into four comparable groups, each of which varied in two parameters: whether or not the mice were administered doxycycline (‘+DOX’ vs ‘no DOX’), or whether allograft cells received a lentiviral expression cassette (‘+hPARG’ vs ‘no hPARG’, i.e. the grafted cells had no cassette). **B** After the cells were allowed to form subcutaneous tumors (≥ 100 mm3) followed by the treatment with doxycycline for 7 days, the expression of hPARG in isolated tumor tissues was confirmed by RT-PCR with two alternative sets of primers complementary to hPARG cDNA (S1 and S2, Table A2 in Supplemental Data section). Doxycycline-dependent increase in hPARG transcript level is expressed as fold increase of hPARG cDNA in doxycycline-treated group (Dox, dark grey bar) over hPARG cDNA level in the tumors derived from animals not treated with doxycycline (Control, light gray bar); *p* < 0.05. **C** The volumes of tumor allografts (mm3) generated by 3 T3 cells lacking the hPARG-expressing cassette (‘3T3 no hPARG’) were measured over indicated time periods (days passed since the cells were grafted). Green line represents average tumor volumes in hPARG-deficient mice exposed to doxycycline (+DOX); blue line represents tumor volumes in hPARG-deficient mice receiving doxycycline-free food (no DOX; *n =* 5 for each time point/experimental group). The doxycycline-treated group developed significantly larger tumors at 3 of the 5 time-points vs control (significance was determined via t-test at each time point; significantly different tumor volumes are indicated by asterisks). Error bars represent SEM in tumor volumes. **D** Similar to the experiment in panel C, the volumes of tumors allografts (mm3) were measured in mice grafted with hPARG-expressing 3 T3 cells. The average volumes were compared at each time point between mice receiving doxycycline (Dox) vs those receiving doxycycline-free food (no DOX). Error bars represent SEM (*n =* 5 at all times indicated). Mice expressing hPARG developed significantly smaller tumors with a delay in tumor onset

To test the effect of hPARG ectopic expression on tumorigenesis, we used an in vivo 3 T3-derived tumor allograft mouse model. The 3 T3 transformation system has led to the discovery of many oncoproteins, including RAS family proteins, human epidermal growth factor receptor 1 (HER1/EGFR) and HER2 [54], and 3 T3 cells form very aggressive subcutaneous tumors that have been used as a standard model for tumor formation and metastases [55–58]. Our experiments compared the 3 T3-derived tumor growth in four tested groups, which were different in regard to the expression of hPARG and the exposure to doxycycline (Fig. 2, panel A).

**Fig. 3:**
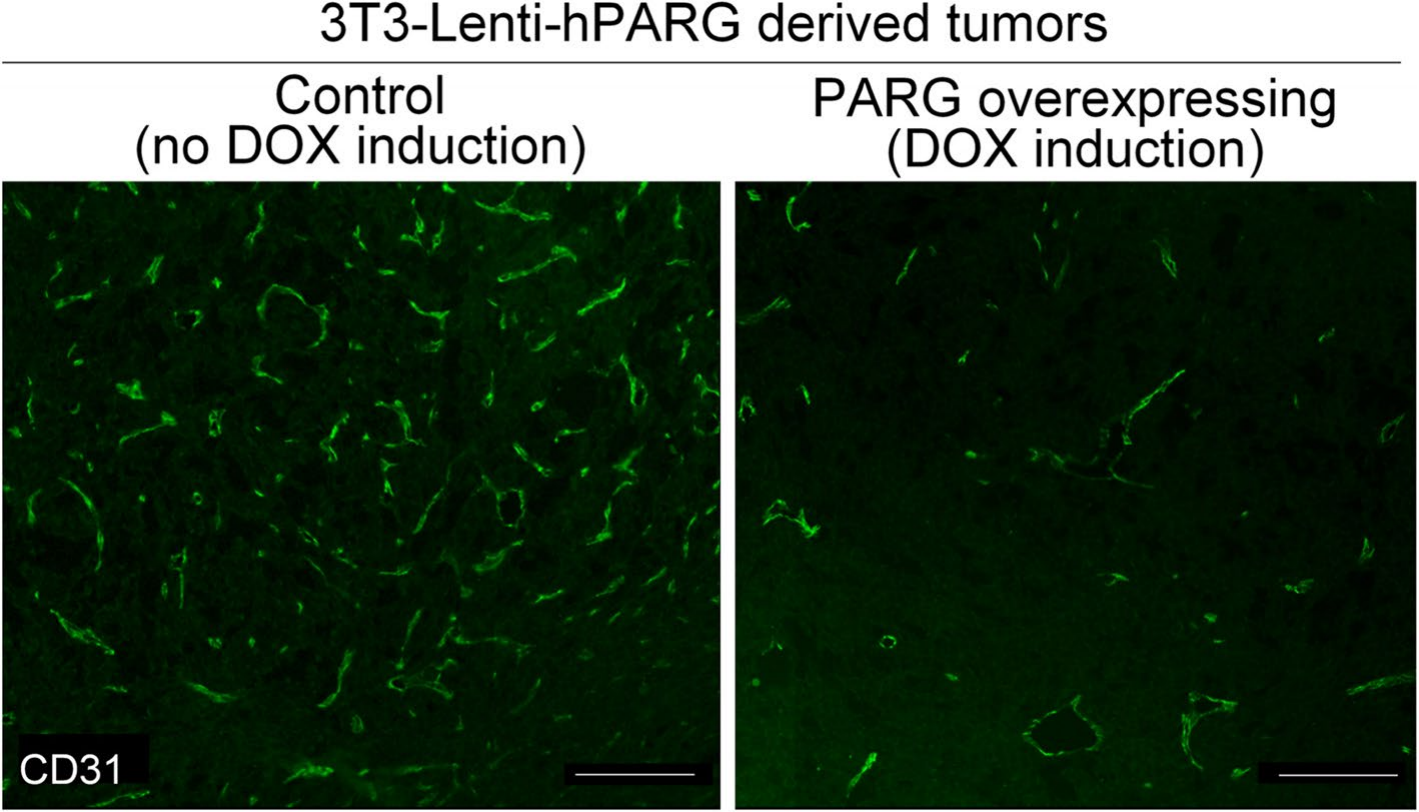
Tumors originated from PARG- overexpressing 3 T3 cells (‘PARG overexpression’) exhibit lower endothelial count than tumors derived from non-induced cells (‘Control’). Tumors were fixed, cryosectioned, and stained with rabbit antibodies to endothelial marker CD31 (BBA7, R&D Systems). The bar represent 100 μm

The expression of hPARG in subcutaneous tumors was confirmed by qPCR (Fig. 2, panel B). 3 T3 cells lacking the hPARG construct produced large tumors both in the presence of and in the absence of doxycycline, with slightly larger tumors in the doxycycline-treated groups (Fig. 2, panel C), which could be attributed for slight, unspecific doxycycline toxicity in immunocompromized animals reported elsewhere [59, 60]. However, hPARG-expressing 3 T3 cells (Fig. 2, panel D) demonstrated a significant reduction in tumor size at all time points with a delay in tumor onset compared to the previous control groups (Fig. 2, panels C) and the group injected with the same cells but given no doxycycline (Fig. 2, panel D, blue graph). Corrected for the slight increase in tumor size due to DOX treatment alone (Fig. 2, panel C, green graph), the true tumor-suppressing effect of PARG overexpression (Fig. 2, panel D) should actually be more dramatic than the apparent effect derived from comparison of two DOX-treated cell lines (Fig. 2, panel D). Remarkably, PARG overexpressing tumors isolated from DOX-treated animals demonstrated significantly lower level of vascularization than tumors isolated from untreated animals (Fig. 3). DOX treatment along without PARG overexpression did not affect angiogenesis (Supplemental Fig. S6).

### The ectopic expression of hPARG in 3 T3 cells changes the expression profile of genes and pathways implicated in tumorigenesis, specifically, chemokines

**Fig. 4:**
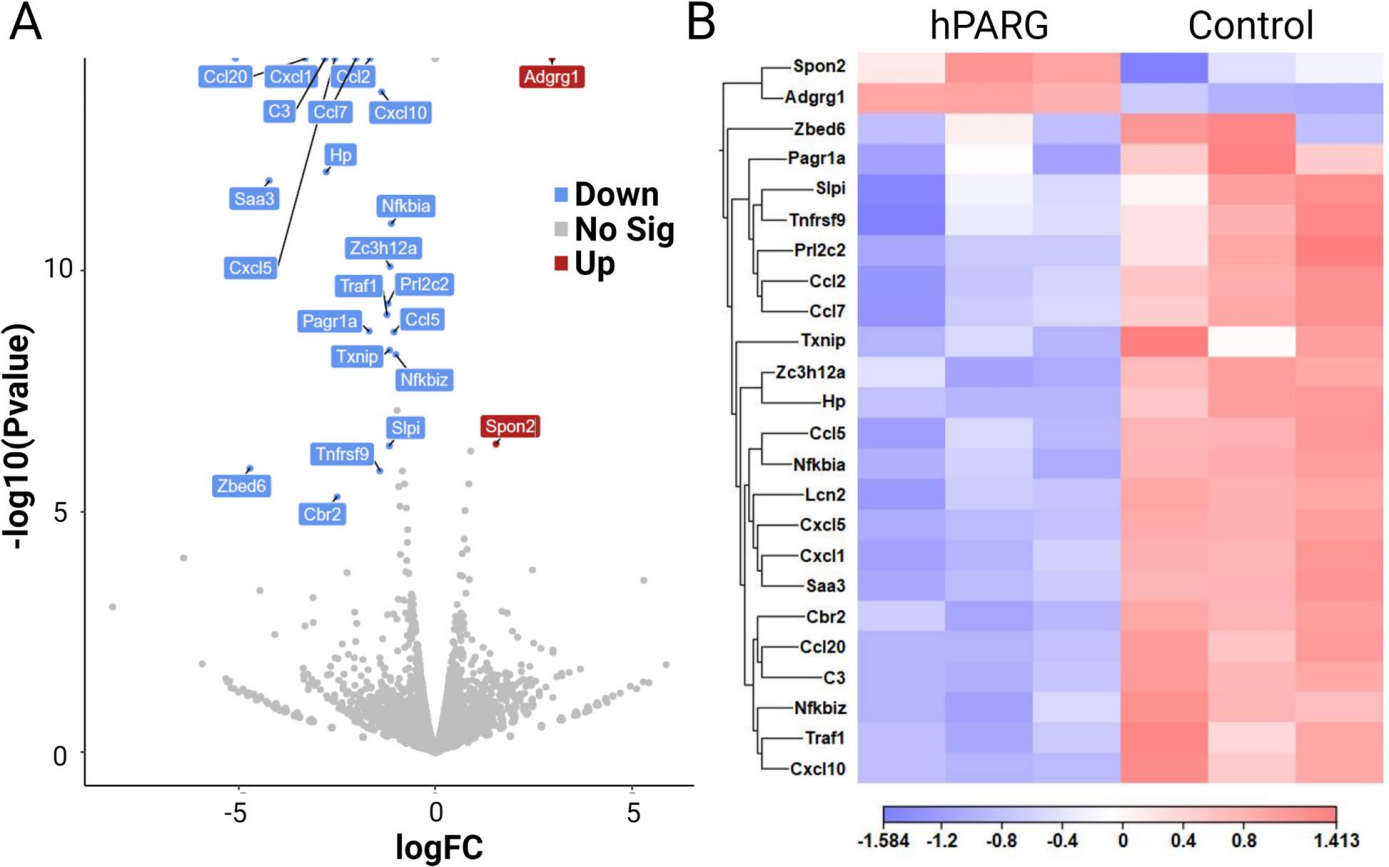
The ectopic expression of hPARG in 3 T3 cells changes the expression profile of genes implicated in tumorigenesis, specifically chemokines. **A** A volcano plot depicting RNA-seq results for fold change of gene expression in cells expressing hPARG vs non-induced control (no doxycycline). Each point represents the average fold change of one gene for three biological replicates of hPARG-expressing cells relative to control. Genes are colored and labeled if they pass the significance value of log2(fold change) > 1 with an FDR corrected *P*-value < 0.05. Upregulated genes are colored red (Up) and annotated with red boxes; downregulated genes (Down) are colored blue and annotated with blue boxes; genes, for which expression level demonstrated no significant change (Not Sig), are represented by unannotated gray dots. **B** Heat map depicting RNA-seq results of expression levels for differentially expressed genes in cells expressing hPARG vs control. Expression levels for each of the three biological replicates from both treatment groups (hPARG vs control) are shown on the X-axis with the corresponding gene names on the Y-axis

As the level of parylation has been shown to affect transcription of multiple genes, we aimed to find which particular genes may change their expression profile due to the increased level of PARG in our cell model. RNA-seq analysis of mouse 3 T3 cells expressing hPARG via doxycycline induction in vitro vs control (no doxycycline) identified 24 differentially expressed genes with greater than 2-fold difference and a false discovery rate (FDR) corrected for *p*-value < 0.05 (Fig. 4 and Supplemental Table A1).

**Fig. 5:**
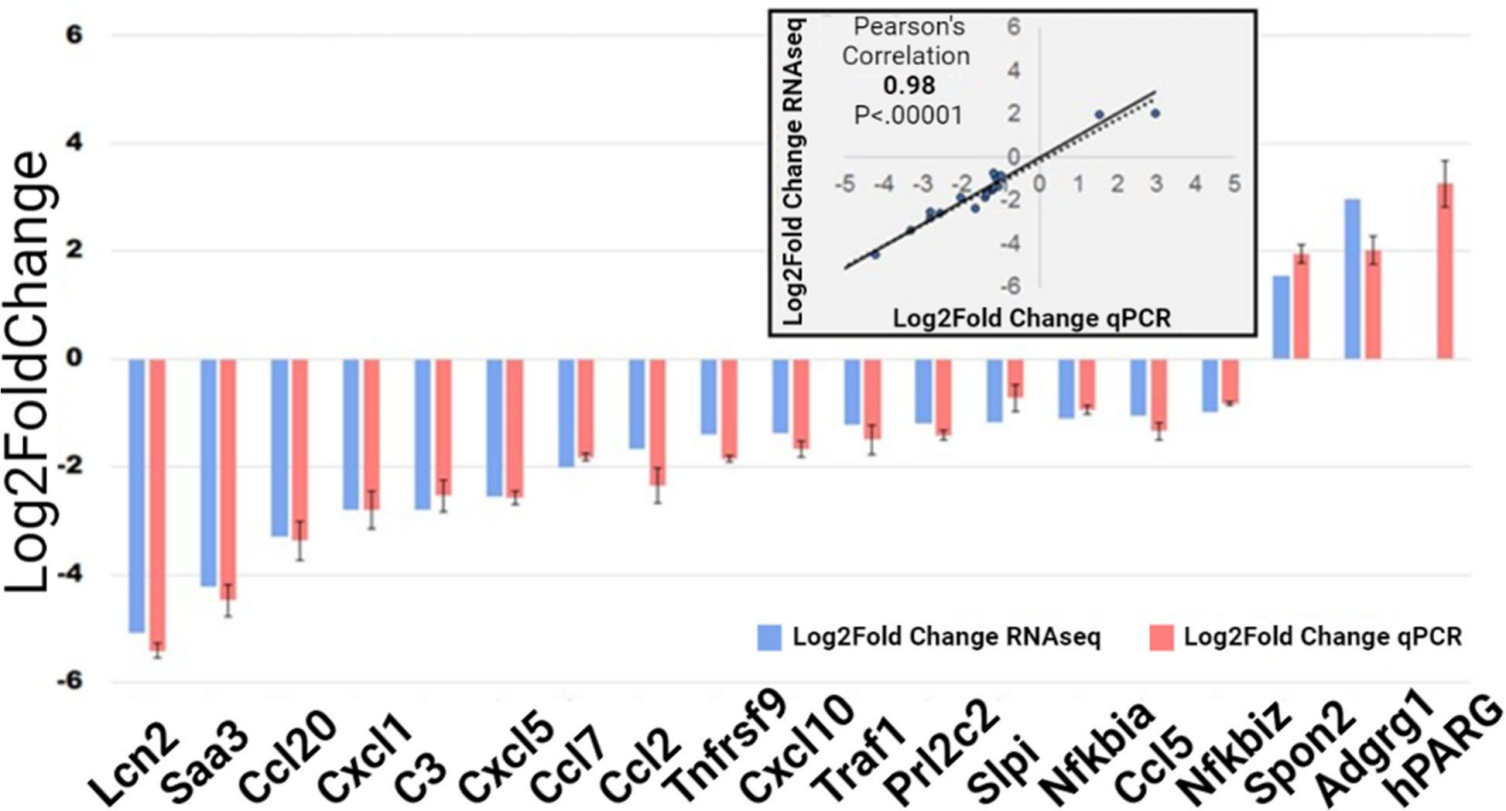
Quantitative PCR (qPCR) experiments confirm that the ectopic expression of hPARG in 3 T3 cells changes the expression profiles of genes implicated in tumorigenesis, specifically chemokines. Differential expression for hPARG+ cells vs control. RNA-seq fold change results are in blue, qPCR fold change is shown in red. Fold change based on 3 biological replicates, error bars for qPCR are in SEM. (insert) Scatterplot of fold change for differentially expressed genes from RNA-seq analyses plotted against qPCR expression fold change results. Pearson’s correlation value of fold change calculated via qPCR and RNA-seq = 0.98 with a *P*-value < 0.00001

Two genes were found to be upregulated in cells expressing hPARG: SPON2 and ADGRG1. The other 22 genes appeared to be downregulated in hPARG expressing cells: C3, CBR2, CCL2, CCL20, CCL5, CCL7, CXCL1, CXCL10, CXCL5, HP, LCN2, NFKBIA, NFKBIZ, PAGR1A, PRL2C2, SAA3, SLPI, TNFRSF9, TRAF1, TXNIP, ZBED6, and ZC3H12A. Expression fold change for genes known to be associated with tumorigenesis was verified via qPCR (Fig. 5 and Supplemental Fig. S2). Log2 fold change of qPCR results vs RNA-seq were plotted and Pearson’s correlation was calculated to be 0.9828 (Fig. 5, insert).

Analysis of our RNA-seq data using PANTHER Statistical Overrepresentation Test (www.pantherdb.org) for the human orthologs of the genes differentially expressed in our study revealed greater than 100-fold cumulative enrichment in the chemokine protein class (CCL5, CCL7, CXCL6, CCL2, CCL20, CXCL10, CXCL2). Among all members of chemokine class family, 3 T3 cells express at elevated level 8 of them and 7 of them were identified as significantly downregulated (Fig. 4**, **Supplemental Fig. S3). PANTHER Overrepresentation Test against the GO molecular function complete dataset found greater than 100-fold enrichment in the expression of factors implicated in binding of chemokine receptors, specifically, the CXCR receptor (CXCL6, CXCL10, /CXCL2), the CCR1 receptor, (CCL5 and CCL7) and the CCR2 receptor (CCL2 and CCL7). PANTHER Overrepresentation Test against the GO-Slim Biological Process dataset found greater than 100-fold enrichment in response to interleukin-1 (CCL5, CCL20, CCL2, CCL7). Finally, bioinformatic analysis by Ingenuity Pathway Analysis (IPA) software (www.ingenuity.com) found 5 genes involved in the interleukin-17A (IL17A) signaling pathway downregulated in 3 T3 cells expressing hPARG (CCL2, CCL7, LCN2, NFKBIA, NFKBIZ), which indicates that hPARG inhibits the IL17A signaling pathway.

## Discussion

PARG is a key tuning factor that regulates the poly(ADP-ribosyl) ation rate and PARP1 dependent genes transcription [5–7]. PARP1 is localized in genes promoters and, by synthesizing pADPr, helps to remove chromatin-associated proteins, denudes the DNA and makes it more accessible for transcription [5]. Noticeably, the main target for PARP1 for poly(ADP-ribosyl) ation is PARP1 itself. When PARP1 becomes heavily automodified, it completely dissociates from chromatin and becomes inactivated. PARG, as the only enzyme that is able to degrade pADPr, plays a unique role in this process and by removing pADPr from PARP1 makes it active again. Therefore, the PARP1-dependent genes transcription relies on adequate PARG functioning and its intensity could be regulated by glycohydrolase level and activity. It was shown in numerous reports that many cancer cells exhibit high level of PARP1 and pADPr, which is frequently cooperate with lower PARG level (Supplemental Fig. S1) [28, 61–67]. It is legitimately suggested that intensive pADPr cycling is needed there to support the enormous division rate. Here we utilized a highly dividing malignant mouse 3 T3 cells that demonstrate high level of pADPr. With the intention to diminish the level of pADPr we used a genetic approach with hPARG overexpression. This gave us a strong advantage of any off-target effects absence, the main problem when chemical inhibition is studied.

The results of our xenograft experiments demonstrate that the elevated level of hPARG in 3 T3 cells reduces their tumorigenic potential in vivo without affecting the cell viability. Comparative RNA-seq analysis of cultured hPARG-expressing cells vs non-transfected cells was utilized to determine the expression of what genes and to what extent might be affected when PARG’s activity is elevated and, consequently, when the cells exhibit decreased tumorigenic activity in vivo. Gene expression fold-change of revealed candidates was validated with targeted RT-PCR and eventually point to the set of genes and pathways specifically regulated by poly(ADP-ribosyl) ation at the early stages of tumor development. Of the 24 genes differentially expressed in hPARG-expressing cells vs control cells (with no hPARG expression), seven belong to the chemokine protein class (CCL5, CCL7, CXCL6, CCL2, CCL20, CXCL10, CXCL2). Multiple publications report that tumors express high levels of chemokines, which recruit cancer-promoting immune cells to the tumor microenvironment, thus augmenting tumor invasion, metastasis, and angiogenesis [68–70]. Notably, other group has published the regulation of CCL2 expression by PARP1/pADPr in breast cancer cells and demonstrated the importance of PARP1 localization in promoter for CCL2 effective transcription [10]. What is also interesting, they found that not all expressed chemokines in studied breast cancer cell line are regulated by PARP1. In our study, we saw the complete synergistic downregulation of all notably expressed chemokines in 3 T3 under hPARG overexpression.

Chemokines are small chemoattractant proteins secreted by different cells to induce and guide the migration, proliferation and differentiation of immune, endothelial, epithelial or other cells that have proper receptors [71, 72]. Cells that are attracted by chemokines follow a signal of increasing chemokine concentration towards the source of the chemokine. The release of chemokines is needed during normal development with extreme importance in blood vessel formation and for wound healing [73–75]. Chemokines also play a major role in immune reactions targeting different immune cells to proper localization and different types of chemokines affect different types of cells [76–78]. Cancer cells utilize numerous chemokines functions to support tumor needs. It was demonstrated, that malignant cells change their chemokine production status that lead to increase endothelial proliferation and tumor vascularization, to attraction of pro-tumorogenic immune cells such as macrophages, neutrophils, lymphocytes, and others [70, 79, 80]. Thus, chemokines could mediate angiogenesis directly, by affecting endothelial cell behavior, or indirectly, via changing tumor microenvironment [81–86].

CCL2 or monocyte chemoattractant protein-1 (MCP-1), which we found to be downregulated 3-fold in cells expressing hPARG (Fig. 4), is known to promote tumorigenesis at all stages of development. Its expression has been shown to induce tumor cell proliferation at the primary tumor sites, stimulate tumor cell migration and invasion into the surrounding extracellular matrix and direct the movement of cancer cells along a chemotactic gradient towards the metastatic site [87]. Targeted gene silencing of CCL2 inhibits triple negative breast cancer progression by blocking cancer stem cell renewal and M2 macrophage recruitment [88]. CCL2 has also been shown to recruit monocytes and monocytic myeloid-derived suppressor cells (MDSCs), which overcome the anti-tumor immune response and promote cancer stemness [70]. Along with CCL7, which is downregulated 2-fold in our hPARG-expressing cells, CCL2 activates the CCR2 receptor (C-C Motif Chemokine Receptor Type 2) pathway, which expression in cancer cells have been demonstrated to promote immune suppression [89]. Furthermore, the product of CCL20 (downregulated 10-fold in hPARG-expressing cells) recruits Th22 cells to the tumor microenvironment and promotes cancer stemness [70]. CCL5 or RANTES (Regulated on Activation, Normal T Cell Expressed and Secreted), which we found to be 4-fold downregulated in hPARG-expressing cells, has been reported to recruit mast cells, T-cells, eosinophils, and monocytes to the tumor microenvironment as well as to promote the proliferation of tumor cells, and to induce tumor cell invasion and migration by upregulating the production of matrix degrading proteins and integrins [90]. Together with CCL7, CCL5 activates chemokine receptor 1 (CCR1), which was found to promote liver cancer by inducing myeloid cell infiltration and angiogenesis [91]. The silencing of CCL7, which was downregulated 2-fold due to PARG’s overexpression, was shown to reduce experimental liver metastasis, whereas CCL5 silencing (downregulated 4-fold in our experiments) reduced metastasis of non-metastatic lung carcinoma cells [92]. The targeted silencing of CXCL1 detected in our search was shown to inhibit the tumor growth in hepatocellular carcinoma [93]. Also, in vivo breast cancer xenografts demonstrated that CXCL1 silencing in tumor-associated macrophages results in a significant reduction in breast cancer growth and metastatic burden [94]. Similarly, of silencing of CXCL1 attenuated the proliferation and radioresistance of glioblastoma cells [95].

The involvement of PARG in suppression of tumor angiogenesis is supported by the observation that, in contrast to its effect on many chemokine genes, hPARG expression upregulates ADGRG1 gene 8-fold. ADGRG1 (Adhesion G Protein-Coupled Receptor G1) encodes G protein-coupled receptor 56 (GPR56) (also known as TM7XN1), regulates adhesion of many cell types and supports stability of various tissues, including normal growth and migration of nerve cells [96–98]. In support of previously published data that the increase in ADGRG1/GPR56 expression suppresses angiogenesis in melanoma tumors [99], our data demonstrate that pADPr pathway positively regulates ADGRG1 in highly proliferative cells, and suggest that hPARG may suppress angiogenesis and contribute to a reduction of tumor size and progression due to its opposite to PARP effect on pADPr moieties and ADGRG1/GPR56 in particular. It also explains the slightly decreased ability of hPARG-expressing cells to heal the ‘wound’ in our wound healing assay (Fig. 1, panels E and F), which may be attributed to the increased adhesiveness of the cells due to the upregulation of ADGRG1 signaling.

One more gene becomes upregulated in 3 T3 overexpressing PARG cells – SPON2 (spondin-2). It is a secreted extracellular matrix protein and a member of F-spondin family. SPON2 is a host innate immunity regulator and an important molecule for efficient microbial response [100]. Positive correlation for SPON2 expression and cancer development, metastasis and invasiveness were shown for different types of tumors, such as ovarian, prostate, colorectal and renal [101–104]. In hepatocellular carcinoma, SPON2 is also upregulated compare to non-transformed tissue, but interestingly, patients with high SPON2 expression have a better overall survival rate. It was shown that SPON2 in this case promotes M1-like macrophage recruitment and inhibits tumor metastasis [105]. The discrepancy of SPON2 expression on tumor behavior could indicate that SPON2 could affect different tumor types in a different ways. Interestingly, SPON2, along with other differently expressed genes in 3 T3 overexpressing PARG, is also a tumor microenvironment regulator, which could indicates this mechanism to underlay the observed effect on 3 T3 derived tumor growth.

It is worth mentioning that, while many of the genes and pathways that we found to be downregulated in hPARG-expressing 3 T3 cells have been shown to promote angiogenesis via upregulation of vascular endothelial growth factor (VEGF) [91, 99, 106], we were surprised to observe no effect of hPARG upregulation on the level of VEGF mRNA. Most likely, because VEGF expression is an immune response to hypoxia in the tumor environment, due to the lack of immune cell components in our in vitro model where the RNA-seq samples were collected, we were not able to detect the effect of hPARG on VEGF. While RNA-seq analysis of tumor tissue samples collected from allografts may confirm PARG-promoted differential expression of VEGF in the follow-up experiments, our model has its own, objective limitations and may not reproduce the gene expression patterns found in various, specific tumors, which further analysis may expand the pool of pADPr-regulated genes. It would also be interesting to see if the induction of PARG expression in the tumors pre-formed in the absence of induced PARG would inhibit further tumor growth and even result in tumor volume reduction (shrinkage).

Among genes downregulated by PARG in our experiments, many genes have been previously described to be driven by either interleukin-1 (IL-1) or interleukin-17A (IL-17A) signaling pathways, specifically, CCL2, CCL7, CCL20, LCN2, NFKBIA, NFKBIZ, CXCL1, CXCL2, CXCL5, CXCL9, and CXCL10 [60–64]. While IL-17A expression is increased in many types of cancer and cancer-associated fibroblasts where it promotes chronic inflammation, tumor cell migration, invasion, and resistance to chemotherapy, and both IL-1 and IL-17A known to be a driver for NFκB-regulated genes and increases chemokines and cytokines in the tumor microenvironment [107], we do not see the direct effect of PARG on interleukin’s expression. It is even more surprising in a light of the reported ability of IL-1 to activate endothelial cells and increase angiogenesis [108]. Based on our data we suggest that pADPr pathway is a downstream regulator of interleukin-dependent factors that lead to the progression of tumors and metastasis [109].

Here would be important to notice that seemingly opposing effects of PARG overexpression and PARG inhibition would be in reality synergistic. When PARG is overrepresented in the system, pADPr is degrading faster and PARP1 could not activate and maintain the transcription of targeted genes. When PARG is inhibited or depleted from the system, PARP1 couldn’t be reactivated from automodifed and dissociated from chromatin state, which leads to the same inability to keep effective transcription of dependent genes. Therefore, results of our study support the anti-tumorigenic effect of PARG depletion or inhibition published by others [110, 111].

**Fig. 6:**
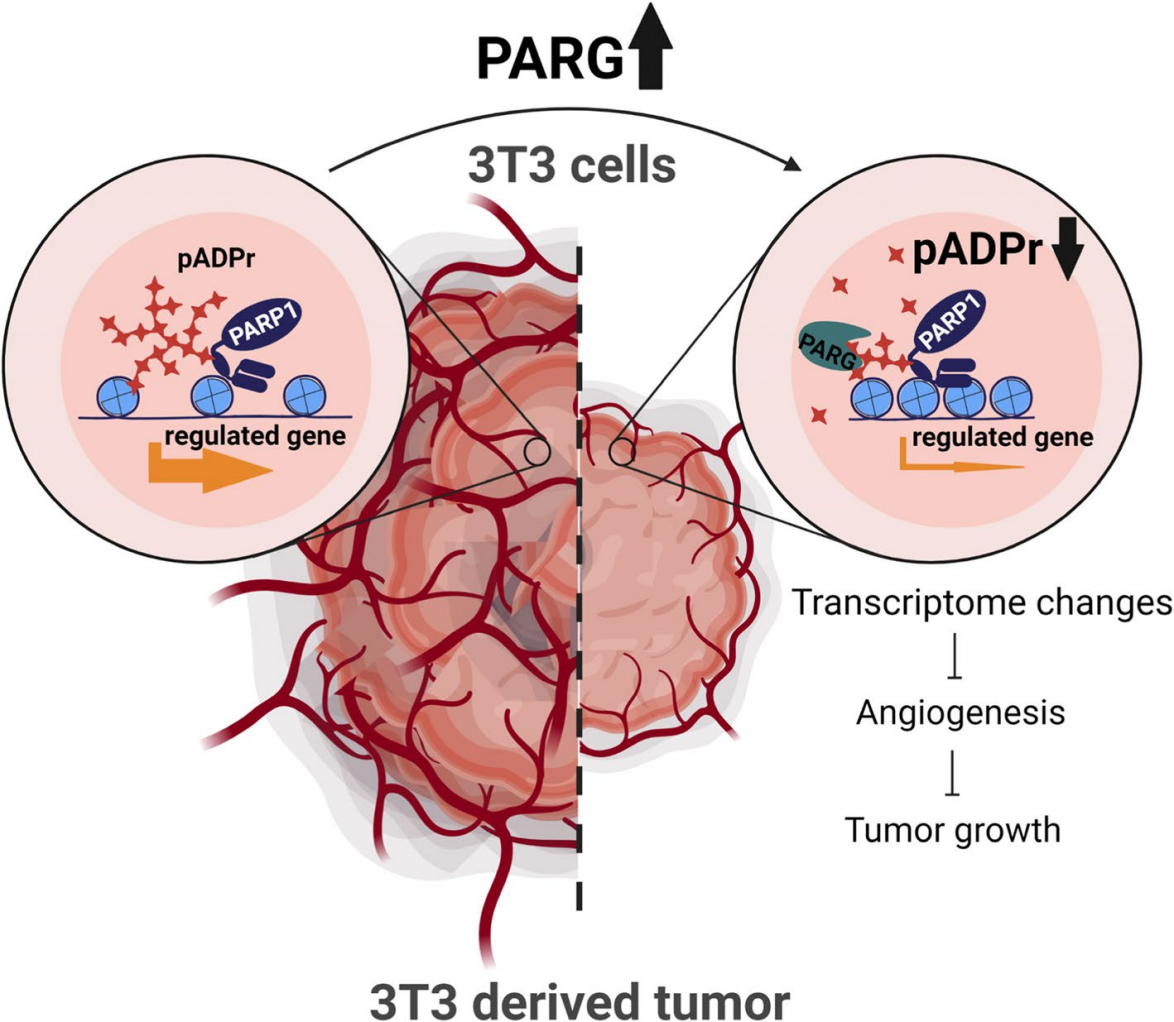
The proposed role of poly(ADP-ribosyl) ation pathway in tumorigenesis. hPARG overexpression in 3 T3 cells cause reduction of pADPr level, transcriptional changes and diminish 3 T3 originated tumor vascularization and growth

In summary, the expression of human PARG in mouse 3 T3 cells leads to changes in gene expression that result in a decrease of the cells’ tumorigenic activity in vivo. These changes occur in the cells before they form solid tumors, pointing on the fact that pADPr pathway controls early events in tumorigenesis. The majority of those changes lead to downregulation of chemokines, broad spectrum, tumor-promoting agents, which may act either through the direct activation of cancer cells, or indirectly, through the activation of tumor-promoting immune cells, cancer-associated fibroblasts or endothelial cells, resulting in inhibition of tumor growth, suppression of angiogenesis and tumor microenvironment (Fig. 6). We did not identified the tumor type originated from 3 T3 cells in our study, but other investigators pointed that it could be closer to carcinoma, than sarcoma type [112]. We studied PARG RNA expression for different cancer types compare to corresponding normal tissue based on TCGA database. Among those types of cancers that are available for this analysis, lower PARG expression was detected for three types of kidney cancer: kidney chromophobe, renal clear cell carcinoma, renal pappilary carcinoma, and for thyroid carcinoma [28]. Malignant glioma, lymphoma, melanoma, renal, prostate and testis cancers were mostly negative for anti-PARG antibody staining as posted on Protein Atlas database. Higher PARG expression were associated with better survival outcome for renal cancer, thyroid cancer and PAM50 Her2 and Luminal B breast cancer subtypes (Supplemental Fig. S1). Previously, we have demonstrated that ccRCC cell exhibit lower level of PARG compare to untransformed cells and PARG overexpression results in the similar anti-tumor effect. While the sets of affected genes were different for these two projects with little overlap, which is pointing on cell type specific variabilities of PARP1 transcription dependency, the main cluster of downregulated genes was also related to angiogenesis term [28]. Therefore, our results are supported by available data that PARG level upregulation could be benefited for several types of cancer.

Due to a quite narrow spectrum of those genes, it would be interesting to further investigate, what makes those genes and/or their regulatory factors a common target for poly(ADP-ribosyl)ation. Based on the results of this work, we propose that, although the crosstalk between PARP and PARG in tumorigenesis and the genetic mechanisms by which poly(ADP-ribosyl) ation exerts its effect on a subset of genes are not yet fully understood, upregulation of PARG’s activity may be a potentially useful and unexplored avenue in the development of cancer treatment. We also acknowledge that the therapeutic, drug-induced overexpression of PARG is unlikely to be achieved. The upregulation of PARG utilized in this work was merely a demonstration of a proof of principle, mimicking the activation of PARG by overproduction of PARG polypeptide. In regard to potential way to activate PARG, it is important to note that both PARP and PARG have been found to have important regulatory phosphorylation sites [11]. Since PARG, unlike PARP, the activity of which is controlled by self-inhibition and resumed by PARG, appears to be the key regulatory enzyme that connects pADPr pathway with other cellular pathways, we intend to use our PARG expression system to search for specific PARG-modifying enzymes, to confirm their role by testing PARG phosphorylation mutants in vivo and in vitro, and to search for their specific, low molecular inhibitors and activators. We predict that this approach would be most effective for solid mass tumors that show high chemokine expression levels, and, as the pADPr pathway happen to be an independent from interleukin-driven, upstream regulation, it would be an auxiliary, independent from anti-inflammatory drugs, intervention [113].

In conclusion, based on the results of this work, we suggest that, although the crosstalk between PARP and PARG in tumorigenesis is not yet fully understood, upregulation of PARG’s activity may be a potentially useful and unexplored avenue in the development of cancer treatment. Specifically, we predict that this approach would be most effective for solid mass tumors that show high chemokine expression levels.

## Supplementary Information

**Additional file 1.**

## Data Availability

All data is available by request to corresponding author. 3 T3-hPARG and non-transfected 3 T3 cells transcriptome analysis is available at GEO, accession number GSE189637. To review GEO accession GSE189637: Go to https://www.ncbi.nlm.nih.gov/geo/query/acc.cgi?acc=GSE189637, Enter token qfezomcozbefjqb.
