# Supplementary material for: PARG suppresses tumorigenesis and downregulates genes controlling angiogenesis, inflammatory response, and immune cell recruitment
Source: BMC Cancer. 2022 May 18;22:557. doi: 10.1186/s12885-022-09651-9 (PMC9118775; doi:10.1186/s12885-022-09651-9)
Supplement: Supplementary file 1 — Additional file 1. [file 12885_2022_9651_MOESM1_ESM.pdf]

SUPPLEMENTAL DATA:

PARG suppresses tumorigenesis and downregulates genes controlling angiogenesis, inflammatory response, and immune cell recruitment

Sarah Johnson1+, Yaroslava Karpova1,2+, Danping Guo1, Atreyi Ghatak1, Dmitriy A. Markov3,* and Alexei V. Tulin1,*

1 Department of Biomedical Sciences, School of Medicine and Health Sciences, University of North Dakota, Grand Forks, ND 58202, USA; sarah.johnson@und.edu (S.J.); danping.guo@und.edu (D.G.); atreyi.ghatak@und.edu (A.G.);. iaroslava.karpova@und.edu (Y.K.);

2 Koltzov Institute of Developmental Biology of Russian Academy of Sciences, Moscow 119334, Russia

3 Department of Cell Biology and Neuroscience, School of Osteopathic Medicine, Rowan University,

Stratford, NJ 08084, USA; markovdm@rowan.edu (D.A.M.)

+ - These authors contributed equally to this work

* Correspondence: markovdm@rowan.edu and alexei.tulin@und.edu

Keywords: PARP; PARG; poly(ADP-ribose) glycohydrolase; poly(ADP-ribose) polymerase; poly(ADP-ribose), chemokines; tumorigenesis; 3T3 cells

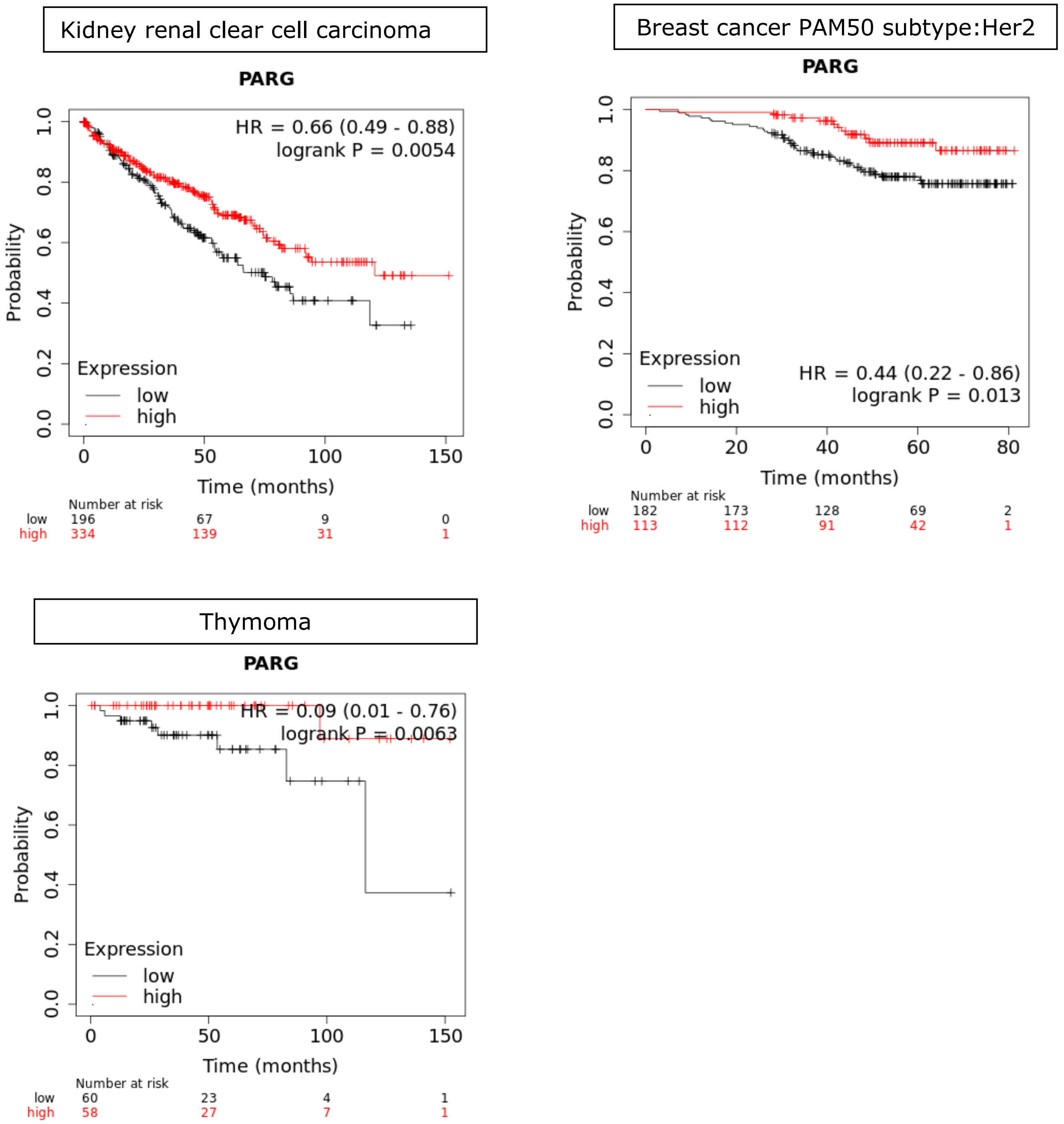

Supplemental Figure S1. PARG expression related survival curves for renal cancer. High expression of PARG is favorable in kidney renal clear cell carcinoma, breast cancer with PAM50 subtype Her2 and thymoma.

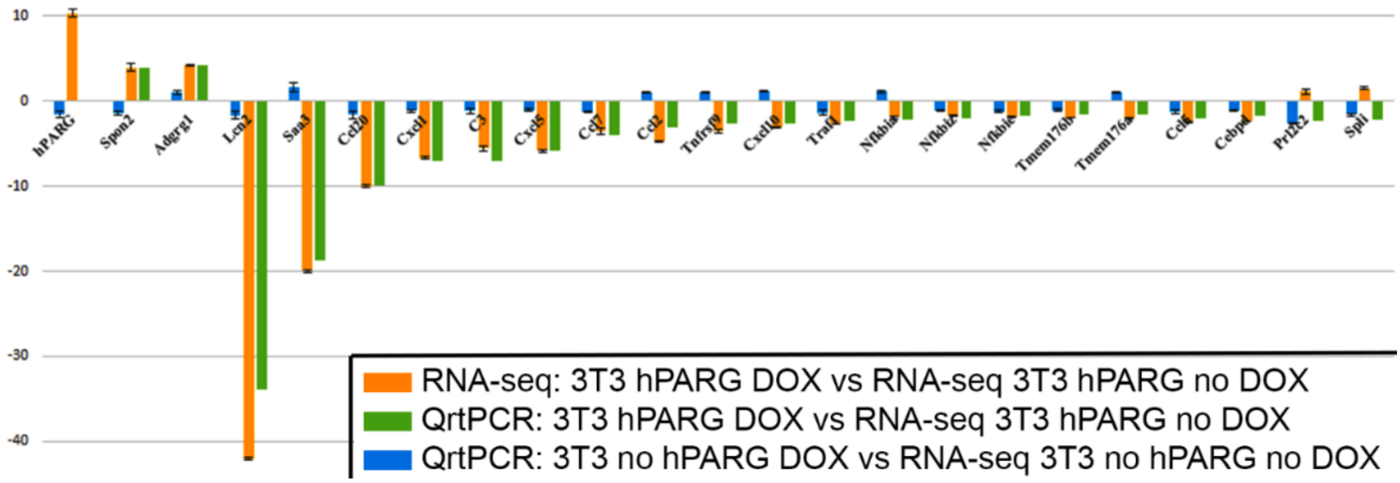

Supplemental Figure S2. Quantitative PCR (qPCR) experiments confirm that the ectopic expression of hPARG in 3T3 swiss albino fibroblasts changes the expression profiles of chemokines. Transduced and non-transduced 3T3 cells, with or without 500 ng/ml Doxycycline, were incubated for 72 hr, then RNA was isolated from the cells and submitted for RNA sequencing. The selected genes were validated by qPCR using specific primers listed in Supplemental Table A1. The colored bars represent the effect of hPARG on differential gene expression in fold change based on 3 biological replicates, error bars for qPCR are in SEM (*p < 0.05). **Green bars:** cells transduced with lentiviral construct expressing hPARG via Doxycycline induction (hPARG +/+ DOX) relative to the same cells without doxycycline (hPARG+/- DOX); blue bars: cells not containing a lentiviral construct were treated with Doxycycline (hPARG -/+ DOX) relative to transduced cells without doxycycline (hPARG -/- DOX); orange bars: cells expressing hPARG via doxycycline induction (hPARG +/+ DOX) relative to the same cells without doxycycline.

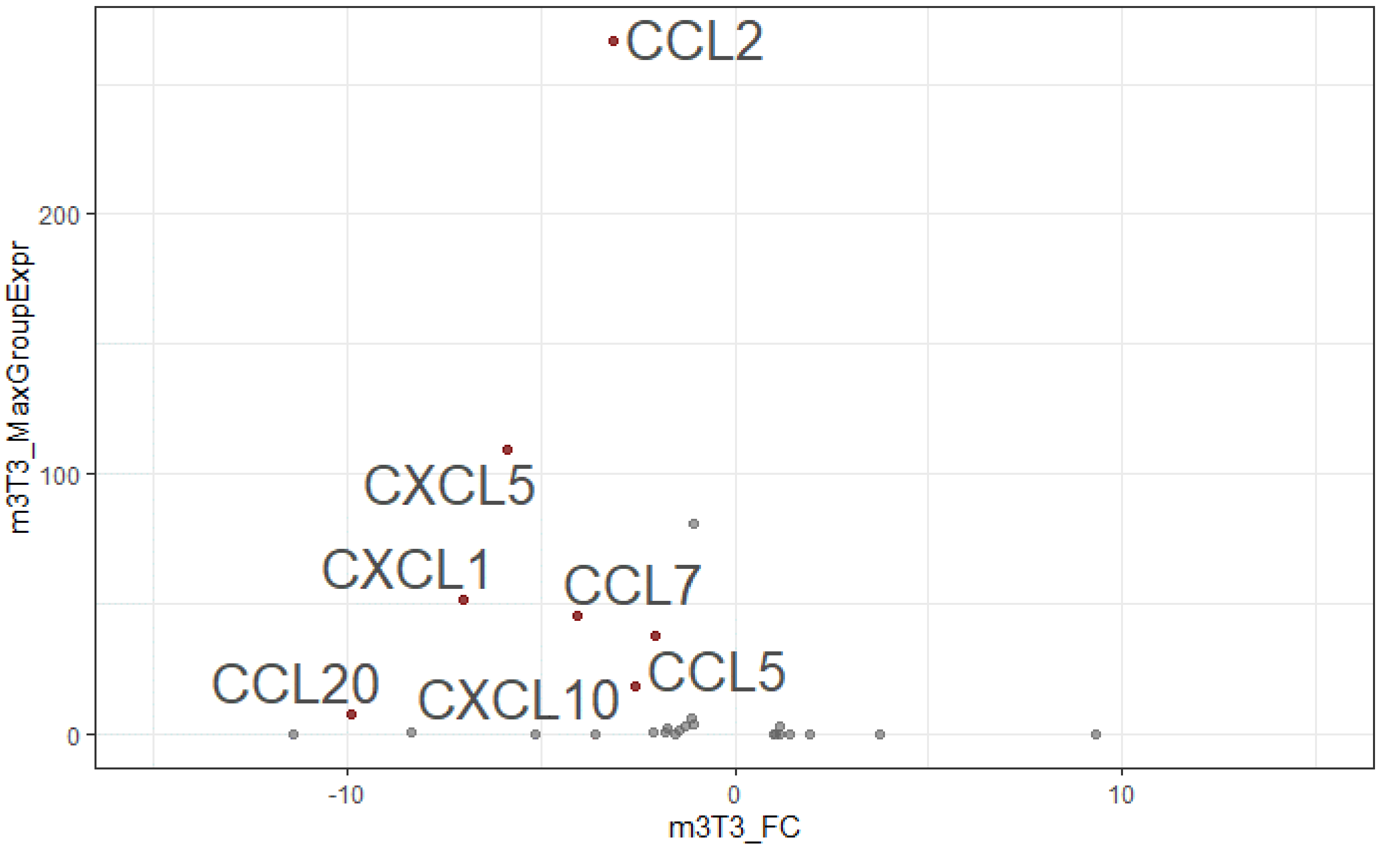

Supplemental Figure S3. All members of chemokine family were plotted relative to expression level (y axis, maximum group expression value, control group for downregulated genes) and fold change (x axis) in PARG

overexpressing malignant 3T3 cells. All chemokines that are expressing in 3T3 cells (expression level more than 1) are become downregulated under PARG overexpression.

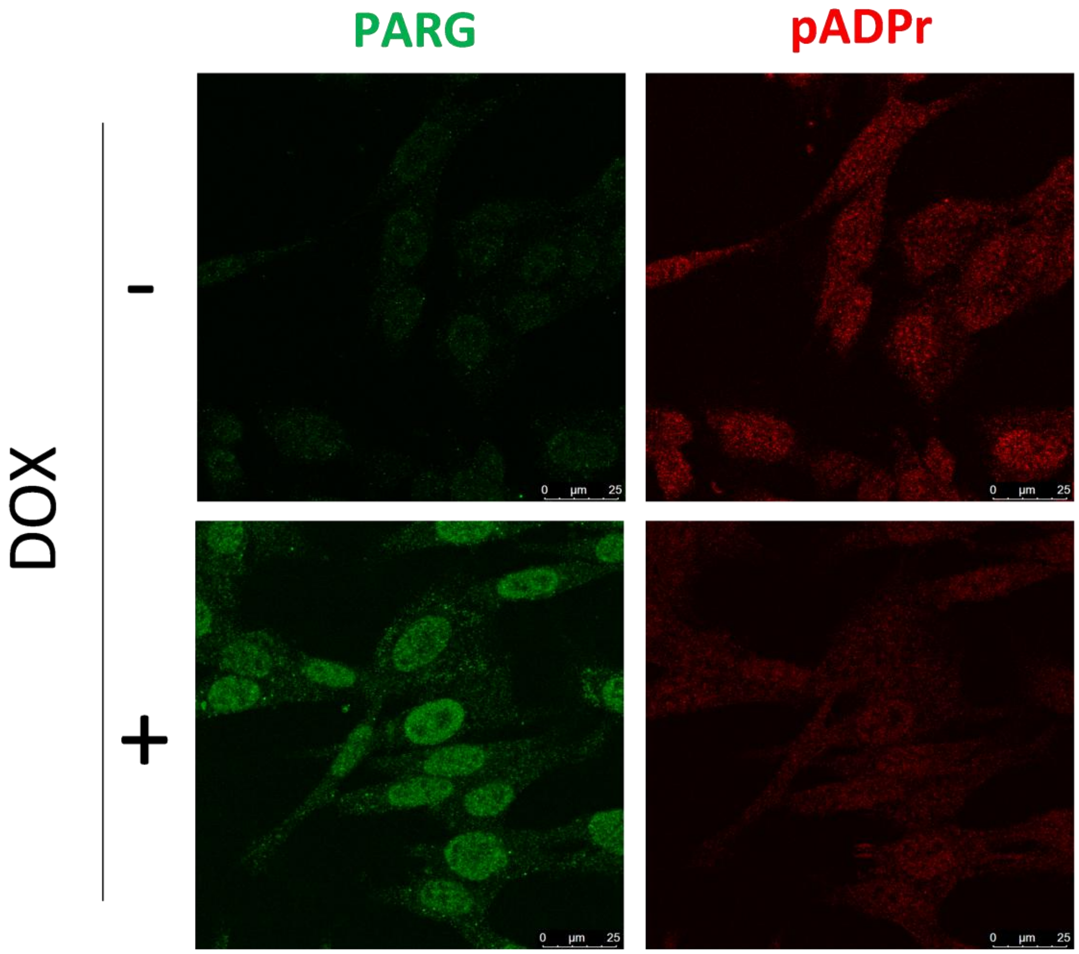

Supplemental Figure S4. PARG overexpression and pADPr reduction in 3T3-hPARG cells. Cells were grown in the presence (+) or in the absence (-) of doxycycline (DOX), fixed, and stained with rabbit anti-hPARG antibodies (green, Cell Signaling) or mouse anti-pADPr antibodies (red, H10, Santa Cruze).

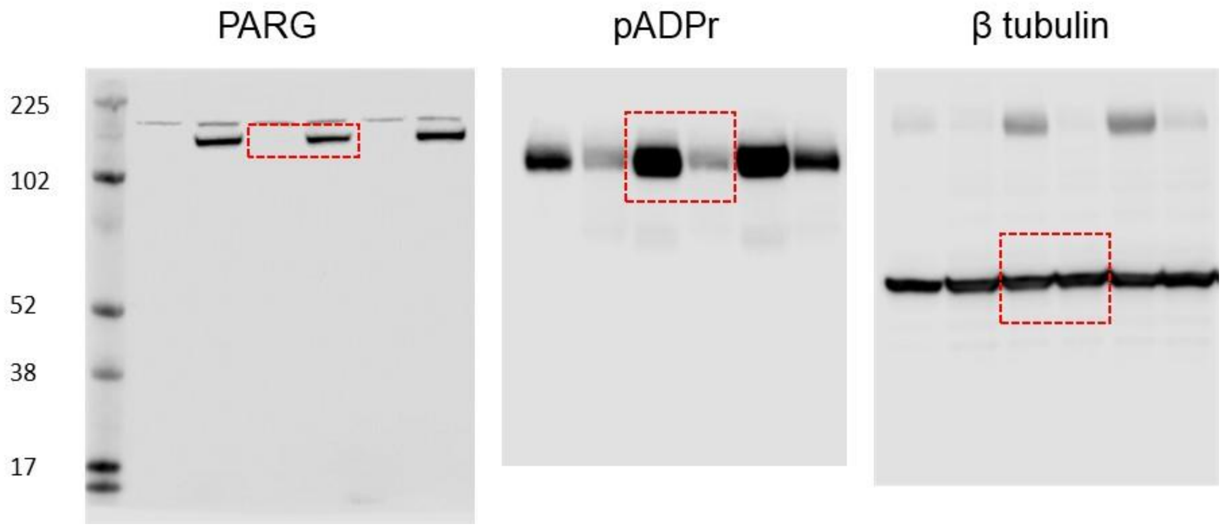

Supplemental Figure S5. Uncropped Western Blots from Figure 1, panel B. Membrane stained with antibodies to pADPr (H10, Santa Cruze), β tubulin (Millipore-Sigma), and hPARG (Cell Signalling).

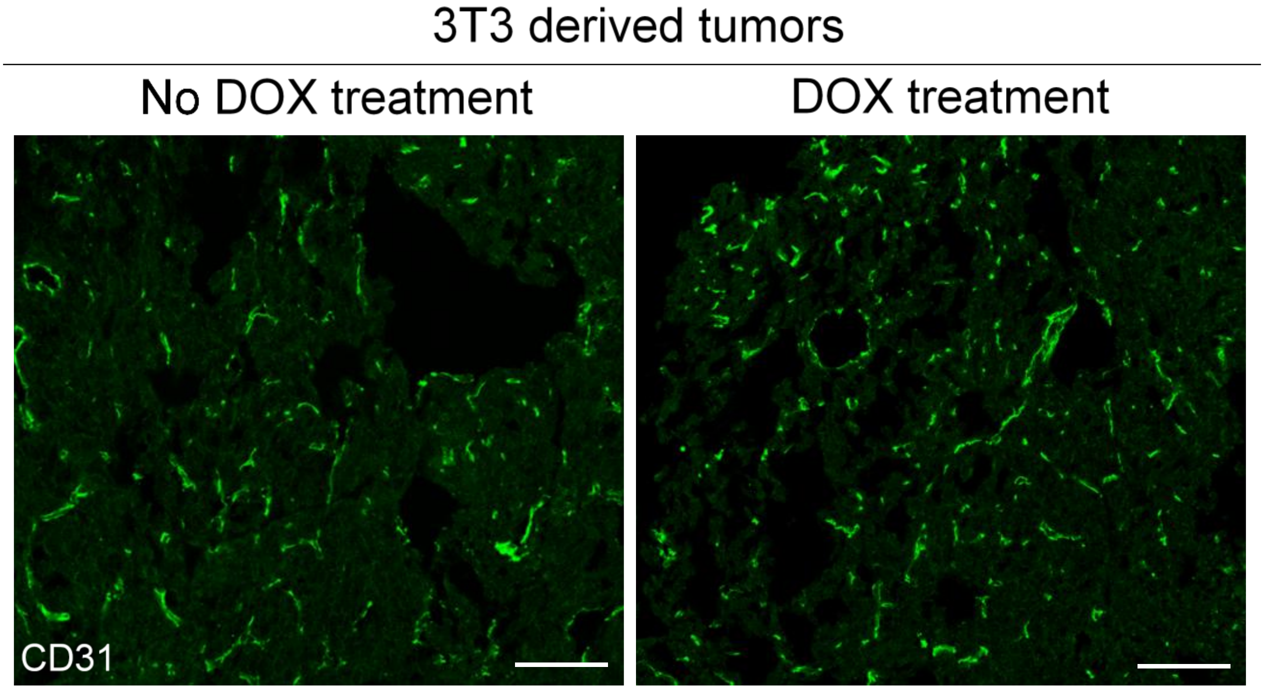

Supplemental Figure S6. Tumors originated from 3T3 cells doesn't change endothelial count under doxycycline treatment. Tumors were fixed, cryosectioned, and stained with rabbit antibodies to endothelial marker CD31 (BBA7, R&D Systems). The bar represent 100 μm.

SUPPLEMENTAL TABLES

Mouse Gene ID	Human Symbol	DIOPT Score	Fold Change qPCR	Fold change RNA-seq	Log fold change RNA-seq	Log fold change qPCR	FDR p-value RNA-seq	Total Counts Mean Control RNA-seq	Total Counts Mean Doxycycline RNA-seq
Adgrg1	ADGRG1	14	4.2	7.83	2.97	2.02	0	23	175.33
C3	C3	14	-5.56	-6.97	-2.8	-2.54	0	144	20
Cbr2	DCXR	4		-5.65	-2.5		6.73E-03	31.67	5.33
Ccl2	CCL2	5	-4.76	-3.15	-1.66	-2.34	0	5,440.00	1,669.33
Ccl20	CCL20	15	-10	-9.87	-3.3	-3.38	0	255.33	25.33
Ccl5	CCL5	13	-2.5	-2.07	-1.05	-1.34	4.55E-06	503.33	236
Ccl7	CCL7	10	-3.57	-4.05	-2.02	-1.82	0	918	218.67
Cxcl1	CXCL2	12	-6.67	-6.99	-2.81	-2.80	0	1,223.33	170.33
Cxcl10	CXCL10	16	-3.13	-2.58	-1.37	-1.67	8.77E-11	689.33	261
Cxcl5	CXCL6	15	-5.88	-5.86	-2.55	-2.57	0	4,302.33	713.33
Hp	HP	12		-6.84	-2.77		3.71E-09	78.33	11
Lcn2	LCN2	16	-50	-33.82	-5.08	-5.42	0	120.33	3.33
Nfkbia	NFKBIA	14	-1.92	-2.17	-1.11	-0.95	3.67E-08	1,597.00	715
Nfkbiz	NFKBIZ	14	-1.79	-2	-1	-0.84	1.23E-05	776.33	375.33
Pagr1a	PAGR1	14		-3.21	-1.68		4.55E-06	394.67	115.33
Prl2c2	PRL	4	-2.6	-2.3	-1.2	-1.42	1.43E-06	662.67	279.67
Saa3	SAA2	7	-20	-18.66	-4.22	-4.48	5.14E-09	59.33	3
Slpi	SLPI	16	-1.6	-2.25	-1.17	-0.73	8.23E-04	642.67	274.67
Spon2	SPON2	14	3.9	2.92	1.55	1.96	7.80E-04	72.33	203
Tnfrsf9	TNFRSF9	14	-3.57	-2.65	-1.41	-1.86	2.29E-03	171.33	62.33
Traf1	TRAF1	15	-2.7	-2.34	-1.22	-1.50	2.33E-06	257.67	107.67
Txnip	TXNIP	15		-2.24	-1.16		1.02E-05	4,776.67	2,093.00
Zbed6	ZBED6	12		-26.26	-4.71		2.18E-03	62.33	2
Zc3h12a	ZC3H12A	14		-2.22	-1.15		2.62E-07	388.33	170

Supplemental Table A1. Differential expression results from RNA-seq, QrtPCR, and DIOPT analyses. Human orthologs for the significantly differentially expresed mouse genes (fold change > 2, FDR corrected p-value < 0.05) found by RNA-seq analyses were determined using DIOPT (DRSC Integrative Ortholog Prediction Tool). DIOPT scores for each ortholog are listed. Fold change of hPARG+/+DOX vs hPARG+/-DOX for each differentially expressed gene is reported for RNA-seq and also QrtPCR (if available). FDR corrected P-values for RNA-seq data are also listed, as well as normalized total mean counts for each treatment group as calculated in CLC.

Genes	Forward Primer	Reverse Primer
Adgrg1	CCGAGCTTCATCTTCTCCTTC	GCTGCTGCAATTCCTTCTTG
C3	GCAGGTCATCAAGTCAGGCT	TAGCTGGTGTTGGGCTTTTC
Ccl2	CTCAGCCAGATGCAGTTAACGCCC	GGTGCTGAAGACCTTAGGGCAGAT
Ccl5	TGAAGATCTCTGCAGCTGCCC	GATTGGAGCACTTGCTGCTGG
Ccl7	CAAAGAAGGGCATGGAAGTCTG	ATCCCTTAGGACCGTGATCAAC
Ccl20	CGACTGTTGCCTCTCGTACA	AGGAGGTTCACAGCCCTTTT
Cxl1	CAAGAACATCCAGAGCTTGAAGGT	GTGGCTATGACTTCGGTTTGG
Cxcl5	GCTGCCCCTTCCTCAGTCAT	CACCGTAGGGCACTGTGGAC
Cxcl10	AAGTGCTGCCGTCATTTTCT	GTGGCAATGATCTCAACACG
Lcn2	ATGTCACCTCCATCCTGGTC	CACACTCACCACCCATTCAG
Nfkbia	TACGAGCAAATGGTGAAGGA	TTCTCTTCGTGGATGATTGC
Nfkbiz	TCTCACTTCGTGACATCACC	GGTTGGTATTTCTGAGGTGGAG
Prl2c2	TGAGGAATGGTCGTTGCTTT	TCTCATGGGGCTTTTGTCTC
Saa3	TGCCATCATTCTTTGCATCTTGA	CCGTGAACTTCTGAACAGCCT
Spli	GCTGTGAGGGTATATGTGGGAAA	CGCCAATGTCAGGGATCAG
Spon2	GCAACTATCCCACAAGACACAG	TGAGGCGTGGGTAGTAGAATG
Tnfrsf9	AGGTGGACAGCCGAACTGTAACAT	TTCTTCTTCCTGTGGACATCGGCA
Traf1	AGATGATGAGGATCGGATCTGT	TTGAAGGAA CAGCCAACACC

Supplemental Table A2. Primer sets used for quantitative real time PCR analysis (3T3 vs 3T3 hPARG).

Sequences are shown in 5′-to-3′ direction.
